# Implementation of a Population-Based Cancer Family History Screening Program for Lynch Syndrome

**DOI:** 10.1177/10732748231175011

**Published:** 2023-05-10

**Authors:** Sayoni Lahiri, Sara Pirzadeh-Miller, Kelsey Moriarty, Nisa Kubiliun

**Affiliations:** 1 Department of Cancer Genetics, 12334 UT Southwestern Medical Center, Dallas, TX, USA; 2 Division of Digestive and Liver Diseases, 12334 UT Southwestern Medical Center, Dallas, TX, USA

**Keywords:** population screening, hereditary cancer, Lynch syndrome, genetic counseling, genetic testing, remote service delivery, patient navigation

## Abstract

**Objectives:**

Lynch syndrome increases risks for colorectal and other cancers. Though published Lynch syndrome cancer risk-management guidelines are effective for risk-reduction, the condition remains under-recognized. The Cancer Genetics Program at an academic medical center implemented a population-based cancer family history screening program, Detecting Unaffected Individuals with Lynch syndrome, to aid in identification of individuals with Lynch syndrome.

**Methods:**

In this retrospective cohort study, simple cancer family history screening questionnaires were used to identify those at risk for Lynch syndrome. Program navigators triaged and educated those who screened positive about hereditary cancer, and genetic counseling and testing services, offering genetic counseling if eligible. Genetic counseling was provided primarily via telephone. Genetic counselors performed hereditary cancer risk assessment and offered genetic testing via hereditary cancer panels to those eligible. Remote service delivery models via telephone genetic counseling and at-home saliva testing were used to increase access to medical genetics services.

**Results:**

This program screened 212,827 individuals, over half of whom were considered underserved, and identified 133 clinically actionable genetic variants associated with hereditary cancer. Of these, 47 (35%) were associated with Lynch syndrome while notably, 70 (53%) were not associated with hereditary colorectal cancer. Of 3,344 patients offered genetic counseling after initial triage, 2,441 (73%) elected to schedule the appointment and 1,775 individuals (73%) completed genetic counseling. Among underserved patients, telephone genetic counseling completion rates were significantly higher than in-person appointment completion rates (*P* < .05). While remote service delivery improved appointment completion rates, challenges with genetic test completion using at-home saliva sample collection kits were observed, with 242 of 1592 individuals (15%) not completing testing.

**Conclusion:**

Population-based cancer family history screening and navigation can help identify individuals with hereditary cancer syndromes across diverse patient populations, but logistics of certain downstream service delivery models can impact outcomes.

## Introduction

### Background and Rationale

It is estimated that there were 149,500 new cases of colorectal cancer (CRC) diagnosed in the United States (U.S.) in 2021, with approximately 52,980 CRC-associated deaths.^1^ Annually, more than 26% ($3.7 billion) of the $14 billion spent on treatment of CRC nationally is spent in the state of Texas alone.^2^ Up to 28% of CRCs in Texas are diagnosed at later stages. Between 2012–2018, Texas ranked 25^th^ out of all 50 U.S. states in CRC mortality, with a higher mortality rate than the national average.^3^

The term, “underserved” refers to patients who experience barriers to receiving health care due to being uninsured, underinsured, and/or geographically isolated. For these individuals, a myriad of obstacles, such as limited economic means, lack of transportation, inability to take time off work, and lower access to childcare, compound existing issues with access to healthcare services.^4-18^ It is therefore unsurprising that underserved patients are at particularly high risk for poor CRC outcomes, including presenting with advanced stage CRC, and worse stage-specific survival.^19,20^

Hereditary CRC can largely be attributed to Lynch syndrome (LS), which is caused by likely pathogenic/pathogenic (LP/P) variants in the mismatch repair genes, *MLH1, MSH2, MSH6, PMS2*, and *EPCAM*.^21^ Compared to the general population, individuals with LS have elevated lifetime risks for CRC, endometrial, ovarian, gastric, and other cancers.^22^

General population CRC screening in the U.S., typically initiated between ages 45–50 years, often fails to identify and prevent LS-associated CRC, which tends to have an earlier age of onset.^23^ Identifying individuals with LS can have significant implications for their healthcare, since cancer risk management for LS includes high-risk surveillance, often starting at younger ages, and/or prophylactic surgery. The evidence for efficacy of CRC surveillance is well-established and necessitated earlier and more frequent colonoscopies for those with LS. Previous data showed a 60% reduction in patients’ CRC lifetime risk, with an extended disease-free lifespan.^24^ However, more recent data points to potential limitations with colonoscopy alone as a means of CRC prevention.^25-27^

With a prevalence of 1:370 individuals, approximately 1,090,666 people in the U.S have a diagnosis of LS, meaning that there would be approximately 78,918 cases in Texas alone.^21^ However, only 2–3% of individuals with LS have been identified, and only 9% of genetic testing for LS has been performed on individuals without a personal history of cancer.^21^ It has also been well-documented that compared to insured or resourced populations, underserved patients are even less likely to have genetic testing that could lead to a diagnosis of LS.^18,28,29^

Population-based screening for LS may help increase the number of LS cases identified.^30-34^ Given that elevated cancer risks associated with LS pose a public health burden and effective interventions exist to reduce morbidity and mortality in individuals with LS, the World Health Organization (WHO) and the Centers for Disease Control and Prevention (CDC) have recommend population-based screening for LS, which has been designated as a Tier 1 Genomic Application by the CDC.^34^ Yet, despite these recommendations, there are a limited number of known large-scale population-based LS screening programs in the U.S.

The dearth of such initiatives prompted an Academic Medical Center (AMC) Cancer Genetics Program (CGP) to implement a population-based cancer family history screening program to increase identification of unaffected individuals with hereditary cancer syndromes, in particular, LS, using grant funding from a state agency, Cancer Prevention and Research Institute of Texas (CPRIT). This program also focused on improving access to genetics services (genetic counseling and testing), particularly in underserved patient populations in North Texas. This program was referred to as Detecting Unaffected Lynch Syndrome (DUAL; CPRIT PP160103).

### Objectives

The primary objectives of DUAL were 1. To promote identification of individuals at risk for LS by implementing a population-based cancer family history screening program in clinics at an AMC and an affiliated county hospital (CH) with a patient population that is primarily underserved; and 2. Improve access to genetic counseling and testing services among underserved patients. Specific aims related to Objective 2 included 1. Utilizing patient navigators to triage and educate patients; and 2. Adopting a remote service delivery model to alleviate barriers that limit access to genetic healthcare for underserved patients.

**Figure 1.**
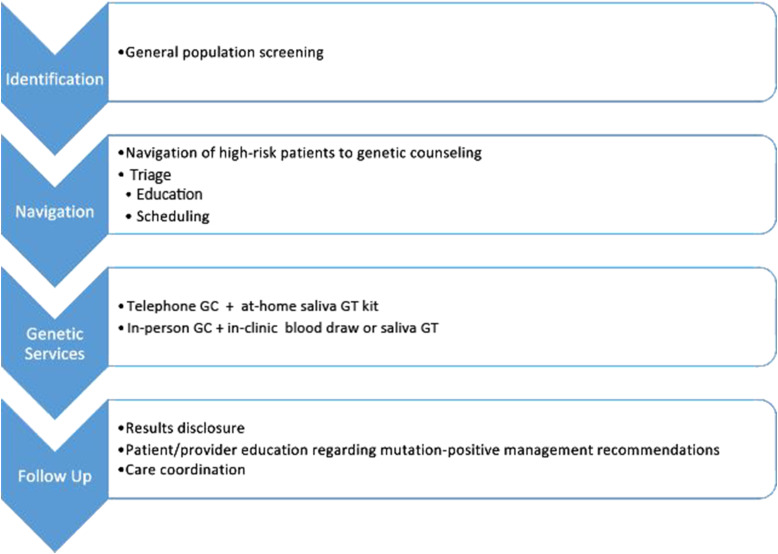
Workflow of the DUAL population-based cancer family history screening and navigation program. GC: Genetic counseling; GT: Genetic testing.

**Figure 2.**
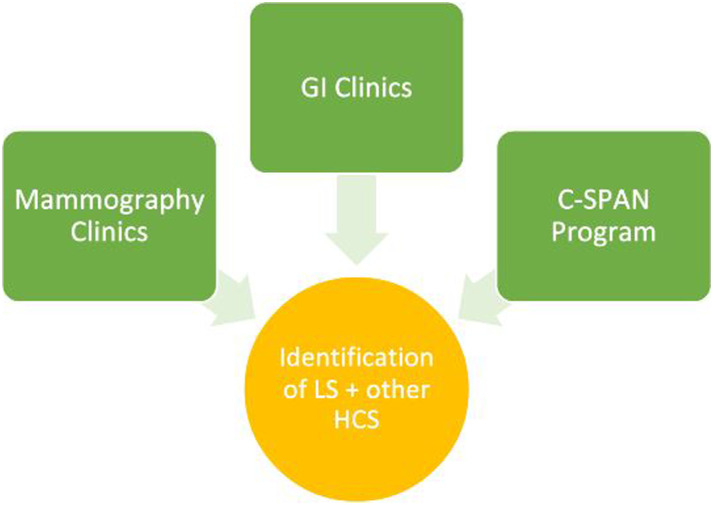
Methods for patient identification utilized for DUAL. C-SPAN: C-SPAN: Partner program that distributes fecal immunochemical tests; GI: Gastroenterology; LS: Lynch syndrome; HCS: Hereditary cancer syndromes.

**Figure 3.**
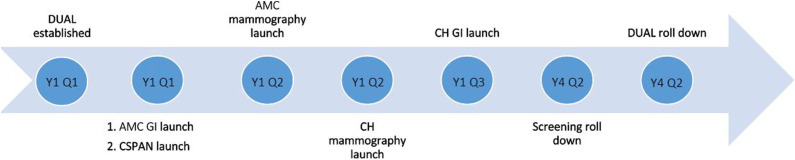
Timeline of DUAL programmatic development. AMC: Academic Medical Center; CH: County hospital; DUAL: Detecting Unaffected Lynch syndrome; C-SPAN: Partner program that distributes fecal immunochemical tests; GI: Gastroenterology; YQ: Year, Quarter.

## Materials and Methods

The reporting of this study conforms to STROBE guidelines.^35^

### Human Subjects Research

The clinical prevention grant-based program was implemented as a clinical quality improvement project not subject to IRB research approval. The aggregate patient data reported in this manuscript falls under UT Southwestern IRB **(**STU 062018-060). This project followed relevant Equator guidelines.

### Program Personnel

The CPRIT grant provided salary funding for 2 full-time genetic counselors, 2 full-time patient navigators, and a full-time genetic counseling assistant, as well as partial salary funding for a grant supervisor and project director to oversee the project.

For DUAL, patient navigators were required to have at least an associate’s degree, but previous navigation experience was not required. The DUAL grant supervisor trained the navigators on strategies for assessing cancer family history and contacting patients to provide education about hereditary cancer and offered clinical services. Navigators worked closely with DUAL genetic counselors to develop an understanding of hereditary cancer, family history-related risks, genetic counseling, and genetic testing.

### Program Design

#### Screening and Navigation

Cancer family history screening was used at multiple clinical sites to identify individuals at increased risk for hereditary cancer syndromes, in particular, LS.

Patients who screened positive based on responses to the family history questionnaires were contacted by patient navigators after chart review to assess more detailed cancer family history (Figure 1). At this touchpoint with the navigator, family history of cancer was confirmed with the patient. If patients met NCCN genetic testing criteria for LS, they were offered an appointment for telephone genetic counseling. Patient navigators also provided education about family history of cancer, LS, genetic counseling, and testing. Patients specifically requesting in-person consultations were accommodated in existing AMC or CH clinics. Some DUAL-eligible patients had already been scheduled for genetic counseling in an existing AMC or CH clinic based on a prior clinician referral for genetic counseling. These patients were seen as part of the DUAL program. All patients scheduled for genetic counseling received telephone appointment reminders.

#### Genetic Counseling and Testing

Hereditary cancer risk assessment was provided by DUAL genetic counselors (Figure 1). Genetic counselors advised patients of the usual risks, benefits, and limitations of genetic testing. Patients were advised that a pathogenic variant could be identified in any of the genes analyzed by testing. Patients were informed of possible preventative screening and/or prophylactic procedures that are generally recommended when hereditary cancer risks are identified, and that any follow-up services would not be covered through the DUAL program. Patients would be referred to specialists either at the AMC or CH, and services would be billed in accordance with their insurance or CH payment plans.

Patients who met NCCN genetic testing criteria for LS (version 2.2016) or Hereditary Breast and Ovarian Cancer syndrome, HBOC (version 1.2016), were offered genetic testing. Consent for testing was obtained initially through mailed paper forms, and subsequently through an online signature platform.^36,37^ Patients who received genetic counseling via telephone were mailed saliva kits for sample collection. The kit contained detailed instructions for sample collection and send-out, and the GCP also sent electronic instructions with accompanying video tutorials to patients with valid email addresses. Patients who did not submit a sample to the laboratory for testing received periodic reminders to return a sample for testing. Reminders were sent electronically or discussed via telephone. Test orders were canceled for patients who did not submit a sample within 90 days of the genetic counseling consultation.

Telephone-based genetic counseling and at-home saliva testing were offered to all patients as concerted efforts to address barriers to care, such as lack of transportation, inability to take time off work, and limited access to childcare services. Additionally, grant funding was used to cover genetic counseling fees for all patients seen through the DUAL program. Grant funding was used to cover the cost of genetic testing for uninsured or underinsured patients.

Over the course of DUAL, test offerings were expanded from an 18-gene hereditary colon cancer panel to larger panels that included up to 83 genes associated with various hereditary cancers (Supplementary Figure 4). All patients who completed genetic testing were at least tested for pathogenic variants in the genes related to LS (*MLH1, MSH2, MSH6, PMS2,* and *EPCAM*). Based on a contract with the genetic testing laboratory, grant funding was used to cover testing costs for panels that analyzed up to 47 genes. Testing costs for patients electing larger panels were either billed to private insurance or the laboratory’s financial assistance program, for uninsured patients.

#### Results Disclosure

Test results were disclosed to all patients via telephone, as per the CGP’s protocol (Figure 1). All patients and designated medical providers were sent copies of the test report as well as a result letter summarizing the test results and genetic counselor recommendations.

Gene-specific resources were also sent to patients who have a LP/P variant, describing their specific gene-related cancer risks and detailed risk-management recommendations. Genetic counselors had thorough discussions with patients who had positive test results about gene-specific cancer risks, cancer risk-management recommendations, and clinical implications for the patient and family members. Patients with positive results were referred to specialists for follow-up as needed. Patients who could not be reached via telephone to disclose results after 3 separate attempts were sent their test report and result letter electronically via the EMR patient messaging portal or by certified mail.

### Setting

Population-based cancer family history screening was implemented in the mammogram and gastroenterology (GI) clinics at the AMC, a private hospital in the United States, not owned by the government, as well as a county hospital (CH) partner, which is owned, maintained, and operated on behalf of the county (Figure 2). The CH provides care to those who are underserved. The CGP focused on Mammogram and GI clinics specifically, because they represent a smaller subset of large population-based clinics such as internal medicine or primary care. The AMC has a catchment area that includes 13 counties in North Texas and encompasses a population of 7.6 million people.^38^ The catchment area of the CH is estimated to have over 2.6 million people.^39^

Family history screening questions were also used to identify patients at increased risk for LS through the C-SPAN program (Figure 2), another CPRIT-funded program (PP1500061). The C-SPAN program distributes fecal immunochemical test (FIT) kits to underserved patients between ages 50–64 in 23 counties in North Texas.

The DUAL program was established in September, 2016 and ended in March, 2020 (Figure 3). Family history screening for LS started in the AMC GI clinic in November, 2016; the AMC Mammography clinic in December, 2016; CH Mammography clinic in February, 2017; and CH GI clinic in April, 2017. Screening was initiated for C-SPAN patients in November, 2016. Family history screening for the DUAL initiative itself rolled down in February, 2020.

### Screening Questionnaires

The family history questionnaires were loosely based on National Comprehensive Cancer Network (NCCN) genetic testing criteria for LS (Version 2.2016) as well family history screeners developed by the CDC.^36,40^ Modifications were made based on stakeholder feedback in the program’s various clinical settings. The modified questionnaires were not validated instruments and were not piloted in our target population. The questionnaires were designed to be less stringent than the NCCN genetic testing criteria for LS so as to account for future changes with the testing criteria themselves, as well as potential changes to the patient-reported family history.

In the mammogram clinics, a family history tool developed by the CDC to screen for Hereditary Breast and Ovarian Cancer syndrome was already in use.^20,40^ This tool was further modified and updated for DUAL to include screening questions about a family history of CRC and endometrial cancer (Supplementary Figure 1). Paper forms were used at the launch of the program, and questions were later embedded into the mammography software at the AMC and the CH so patient responses could be captured electronically by mammography technicians. This allowed for subsequent development of an algorithm within the reporting structure of the mammography software that generated automated lists of screen-positive patients for the patient navigators to contact (Supplementary Figure 2). GI clinic leadership had concerns about the length of the proposed family history tool, and its impact on length of the GI visit. Due to unique needs in the GI clinics at both hospitals, a separate LS screening tool focusing on personal and/or family history of CRC and endometrial cancer only (Supplementary Figure 3), was built into the smart forms within the electronic medical record (EMR), used by healthcare providers. An EMR-based algorithm was created to automate identification of screen-positive patients, who then populated a patient registry housed in the EMR. The GI clinic LS screening tool was replicated and embedded in the C-SPAN software as well, and medical assistants for C-SPAN reviewed eligible patients.

### Program Participants

All adult male and female patients, age 18 and above, attending a screening mammogram appointment in the mammography clinic at the AMC or the CH were eligible to complete the cancer family history screening questions. All adult male and female patients, age 18 and above, attending a screening colonoscopy appointment or consulting with a clinician in the GI clinic at the AMC or the CH were also eligible for screening. All patients enrolled at the CH were considered underserved for the DUAL program. C-SPAN patients who had a positive FIT result and were already scheduled for a follow-up colonoscopy were eligible for screening. DUAL patient navigators followed-up with all eligible screen-positive patients as outlined in the program design.

Patients were eligible to participate in DUAL until the end of the grant-funded time period and the number of patients enrolled was not set prior to the launch of DUAL.

Demographics of the AMC and CH patient populations respectively that were focused on for DUAL include the following ethnic distributions: White 51% and 16%; Black or African American 17% and 26%; Hispanic or Latino 22% and 49%; Asian 4% and 3%; and Other/Unknown 6% and 5%. Demographic data for the C-SPAN patients were not made available to the DUAL program. As these patients were ascertained through cancer screening clinics and programs, patients are considered healthy individuals pursuing evidence-based cancer screening.

### Data Management

Variables that were tracked over the course of the study included number of patients screened; screen-positive rate; number of patients navigated and navigation outcomes; number of patients scheduled for genetic counseling; genetic counseling appointment and genetic testing uptake; test complete rates; and genetic testing results. As this was not a research study, exposures, predictors, confounders, and effect modifiers were not evaluated and special measures were not put in place to address potential bias.

Data regarding number of patients screened and number of screen-positive patients were acquired from the AMC and CH mammography software programs, the EMR, and the C-SPAN software, respectively. Navigation outcomes data as well as data on genetic counseling and testing outcomes were tracked using the CancerGene Connect (CGC) navigation platform. CGC is a web-based program that combines the collection of family and medical history, cancer risk assessment, psychosocial assessment, report templates, a result tracking system, and a patient follow-up system. CGC also has a navigation tracking platform.^41^ DUAL program metrics and outcomes were tracked using CGC data reports (IRB *STU 062018-060*).

Genetic counseling and testing uptake and completion rates were calculated from CGC data reports. Descriptive statistics were used to report quantitative data. Genetic testing completion rates were compared between patients considered to be underserved and those not considered to be underserved. Chi-squared analysis with a p-value of .05 was used to compare genetic counseling completion rates for DUAL patients from the CH who had scheduled telephone appointments to the genetic counseling completion rates for non-DUAL patients who had in-person genetic counseling appointments at the CH during the same time period.

## Results

### Identification Through Cancer Family History Screening and Patient Navigation

**Figure 4.**
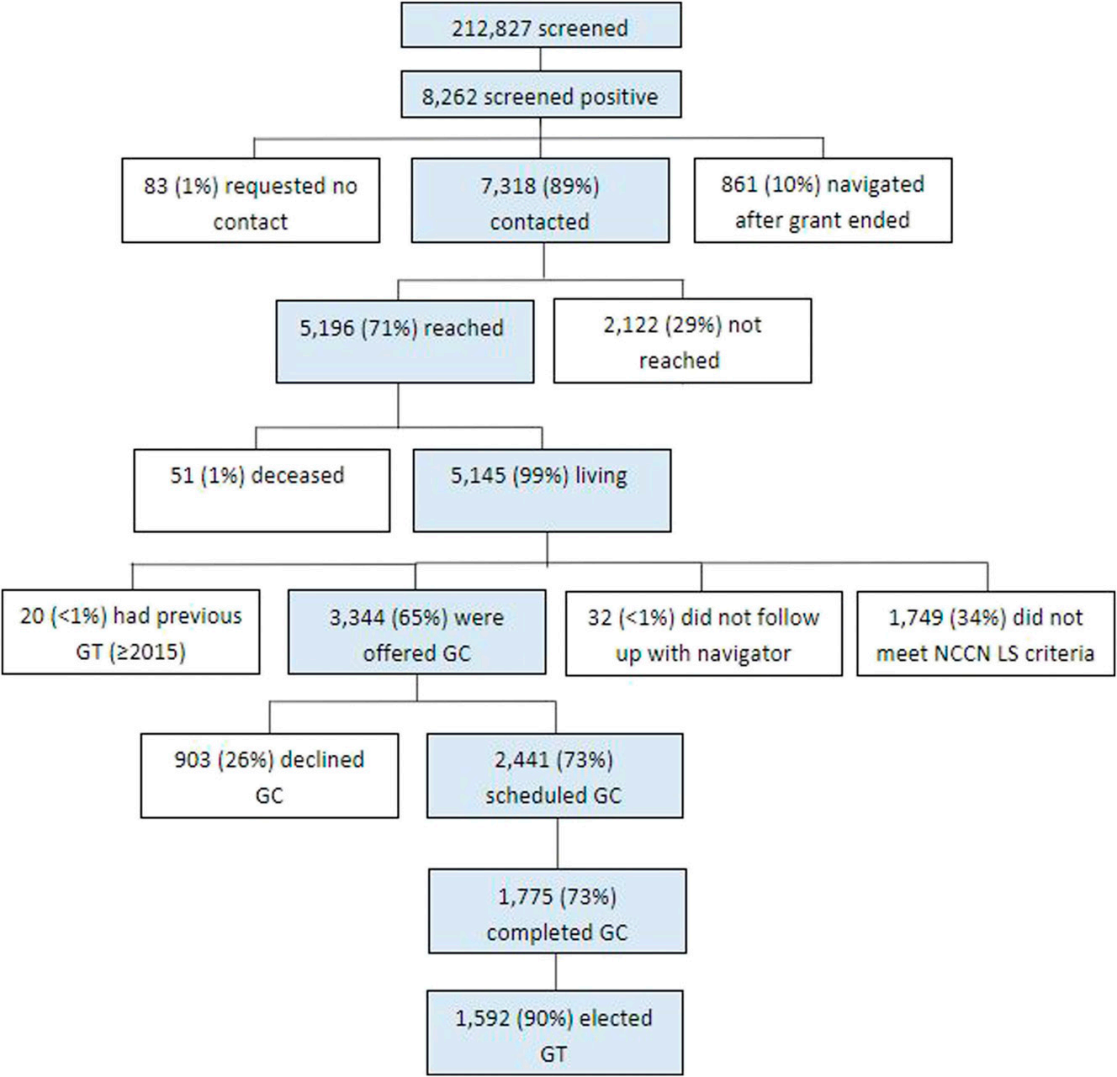
DUAL screening and navigation programmatic outcomes. DUAL: Detecting Unaffected Lynch syndrome; GC: Genetic counseling; GT: Genetic testing; NCCN LS criteria: Lynch syndrome evaluation criteria in Genetic/Familial High-Risk Assessment: Colorectal version 2.2016 by National Comprehensive Cancer Network®.

Over the life of the DUAL program, 169,607 mammography patients, 42,320 GI patients, and 900 C-SPAN patients were screened for a total of 212,827 patients who underwent family history screening to identify individuals at increased risk for LS. Over half of the patients in the GI (22,502/42,320; 53.2%) and mammography (91,249/169,607; 53.8%) clinics were underserved patients from the CH. We observed a 3.9% (8,262/212,827) screen-positive rate. Navigators were able to reach 70.3% (5,145/7,318) of the patients they attempted to contact, of whom 65% (3,344/5,145) met NCCN testing criteria for LS upon confirmation of family history and were offered genetic counseling. Seventy three percent (2,441/3,344) of patients who were offered a genetic counseling appointment elected to schedule the appointment (Figure 4).

The overall genetic counseling appointment completion rate was 73% (1,775/2,441) of those scheduled after being screened at a clinical site, and then navigated to genetic counseling services by a program navigator. This is compared to a genetic counseling appointment attendance rate of 26–31% that has been reported in the literature.^42,43^ Internal, unpublished GCP data from other cancer prevention initiatives shows that involvement of patient navigators led to genetic counseling appointment attendance rates of approximately 50%–59%. Specifically, among the underserved patients who were scheduled for a telephone consultation, the appointment completion rate was 78% (683/876). The appointment completion rate for non-DUAL patients who had in-person genetic counseling appointments scheduled at the CH for the same time period was 51.2% (2,595/5,070). As such, DUAL patients who had telephone appointments had a significantly higher appointment completion rate, *X*^*2*^ (1, N = 5946) = 215.5, *P* < .05. The majority of those who underwent genetic counseling also elected to proceed with genetic testing (1,592/1,775, 89.7%) and completed the testing process (1,350/1,592, 84.8%).

Reasons for not undergoing genetic testing included not meeting NCCN genetic testing criteria for LS or HBOC (38/183, 21.0%), no interest in testing (136/183, 74.3%), and previous genetic testing (9/183, 4.9%). As previously mentioned, the family history screening questionnaires were less stringent than NCCN genetic testing criteria for LS. As a result, genetic testing may not have been medically indicated for all patients after thorough review of the family history with a genetic counselor. Additionally, in some instances, the initial family history reported by the patient was different from what was reported to the genetic counselor, which also led to some patients not meeting NCCN genetic testing criteria for LS or HBOC.

Among those who initially elected genetic testing, 15.2% (242/1,592) did not complete the testing process, largely because they did not submit a sample for testing (199/242, 82.2%) or because there were logistical issues with the first sample provided (32/242, 13.2%) and subsequent samples were either not provided or also had logistical issues. Logistical issues included sample failures, mislabeled/unlabeled sample tubes, or leaking sample tubes. Underserved patients had a higher rate of incomplete genetic tests due to a logistical issue with the sample (25/32, 78.1%) or due to failure to send a sample for testing (125/199, 62%). While some patients initially elected to proceed with genetic testing, they later contacted the clinic and requested their test be canceled (11/242, 4.5%).

In total, 12.1% (164/1,350) of patients who completed genetic testing were found to have a LP/P variant associated with hereditary cancer. Not all LP/P variants identified indicate a need for altered clinical management for the patient (not clinically actionable). However, for LP/P variants in several of the genes identified via genetic testing, evidence-based guidelines (eg NCCN, United States Preventative Services Task Force) or expert recommendations for cancer surveillance, and/or prophylactic measures are in place due to a demonstrated impact on cancer prevention (clinically actionable). The observed rate of such clinically actionable LP/P variants was 9.9% (133/1350). This constitutes 81.1% (133/164) of the total LP/P variants detected. Among the patients with clinically actionable LP/P variants, 47 (47/133, 35.3%) were associated with LS, *MLH1, MSH2, MSH6,* and *PMS2 (Group 1).* Among this group, 3 patients had 2 clinically actionable LP/P variants identified, *MLH1/APC, MLH1/PMS2,* and *PMS2/HOXB13.* The overall yield of LS was 3.48% (47/1,350) among patients who completed testing. Sixteen patients (16/133, 12.3%) were found to have LP/P variants in other CRC-associated genes: *APC, AXIN2, BMPRA1, SMAD4,* and homozygous *MUTYH* (Group 2). The remaining patients (70/133, 52.6%) were found to have LP/P variants in genes not typically associated with hereditary colorectal cancer and included the following: *AIP, ATM, BARD1, BRCA1, BRCA2, BRIP1, CDKN2A, CHEK2, FH, FLCN, HOXB13, MITF, NBN, NF1, PALB2, PTEN, RAD50, RAD51 C, RAD51D, SDHD, TP53,* and *TSC2 (Group 3)*. This group included a patient with 2 clinically actionable LP/P variants, *BMPR1A/RAD50*. Pathogenic variants in the genes in Groups 2 and 3 are associated with a spectrum of various different cancers. The level of cancer risk associated with the individual variant and the degree of cancer risk-reduction conferred by preventative measures varies by gene. Though patients in Groups 2 and 3 (86/133, 64.6%) did not have Lynch syndrome, they had LP/P variants identified in other genes that impact clinical cancer risk management per NCCN or other consensus guidelines, requiring prophylactic surgery and/or high-risk surveillance.

### Discussion: Successes and Lessons Learned

The DUAL program as a whole demonstrated several successful outcomes as well as opportunities for improvement. DUAL, a unique and population-based clinical cancer prevention program, allowed the CGP to implement cancer family history screening across clinical sites to systematically screen a large volume of patients and aid in the identification of patients with clinically actionable LP/P variants associated with LS.

Comparison of this cancer family history screening program to other large-scale approaches for identification of LS, such as screening all CRC and endometrial cancers for MMR protein expression, show certain benefits and limitations. Benefits include ability to identify individuals with LS (and potentially other hereditary cancer syndromes) prior to a cancer diagnosis; improving the opportunities for cancer prevention; the economic savings to the individual, family, and even society that stem from cancer prevention (eg elimination of treatment related costs); and the need for less institutional funds and resources to implement a screening questionnaire compared to a tumor screening program. Limitations include lower sensitivity and yield, potential for inaccurate reporting of family history, and patient dropout throughout different steps of the screening and navigation process.

Though the initial intent of the DUAL program was to identify individuals with Lynch syndrome, expanded test offerings (from a CRC-focused genetic testing panel to a larger pan-cancer panel) eventually led to the identification of a significant number of secondary findings (LP/P variants in genes not typically associated with hereditary colorectal cancer) in the DUAL patients, who otherwise may not have known about their cancer predisposition or been aware of preventative measures. The observed rate of secondary findings supports existing literature that cites decreased sensitivity of national guidelines related to larger cancer panels, which has implications for ongoing cancer risk-management needs for the patient and healthcare system.^44-49^

To the authors’ knowledge, there are no other large-scale hereditary cancer family history screening efforts that have captured a greater number of at-risk patients. Furthermore, this work represents the largest known hereditary cancer risk identification effort in a primarily underserved population. The DUAL program shows the potential impact of true population screening for cancer family history across different socioeconomic populations.

Patient navigation is a process-based system organized to (1) identify cases, (2) identify and address barriers to care, (3) implement a specific care plan, and (4) measure effectiveness by tracking cases through to specific outcomes.^50^ While data surrounding patient navigation for genetics services is scarce, 1 randomized trial showed patient navigation improves uptake of cancer genetic counseling services in an insured population by 13%.^51^ DUAL data shows a 73% appointment attendance rate among patients initially identified through cancer family history screening. The higher uptake of genetic counseling compared to what has been cited in the literature suggests that use of patient navigators and remote service delivery models adopted by the program were critical to increased uptake of genetics services on a large scale.

With DUAL, an emphasis was placed on improving healthcare access to underserved patients, and we observed significantly improved uptake of genetic counseling services using telephone-based genetic counseling in the underserved population. This service delivery model was anticipated to be effective given many individuals (89%) report using a phone for medical discussions.^52^ Studies in underserved populations have noted that internet access through mobile technologies has increased access to healthcare information.^53,54^ National surveys found 91% of families living below the poverty level have some type of internet access, and of Americans earning <$30,000 annually, 71% owned a smartphone making telephone-based services reasonable for this subset of the population.^55-57^ Furthermore, review of 2019 internal program data revealed 84–88% of safety-net hospital clinic uninsured/Medicaid patients provided an email address and reported internet connectivity.

Additionally, the literature shows that when indigent patients are offered the option of telephone or video genetic counseling, they preferred the former and had an increased uptake of genetic counseling with the telephone service delivery model compared to other interventions.^58,59^ In DUAL, we observed improved telephone genetic counseling completion rates compared to in-person genetic counseling among underserved patients. This finding was also observed in another study recently published by the CGP,^60^ with a statistically significant increase in completion of scheduled telephone genetic counseling appointments vs in-person. Using a telephone-based service delivery model supported a major goal of this project to increase access to genetics services, especially among underserved populations. Our programmatic data builds an evidence-base to support telephone-driven genetic counseling services as an access point across populations. Telephone-based services are more accessible for underserved patient populations, who may experience barriers accessing video-based services, which require web networks and costlier internet access with data usage.

The caveat to offering remote clinical services was observed with lower completion rates for genetic testing, While uptake of genetic counseling visits via telephone was a success, a challenge encountered with remote service delivery was a higher saliva sample failure rate and no sample rate in the underserved population, which translated to lower genetic testing completion rates and a missed opportunity to identify individuals with clinically actionable LP/P variants associated with hereditary cancer. The same resources (written and video-based sample collection and send-out instructions as well as sample submission reminders) were provided to underserved patients and non-underserved patients alike. However, lower test completion rates among underserved patients suggests the need for additional investment in resources such as further education or assistance in providing a saliva sample, or even allowing for alternate methods for sample collection (ie, mobile phlebotomy). Further study, perhaps through qualitative research to query participants from different socioeconomic populations, needs to be undertaken to continue investigating barriers and determine if there is need for different versions of education between populations to help ameliorate this issue.

Some of the biggest implementation challenges for DUAL stemmed from the modification of existing hereditary cancer screening programs across different clinical sites, each with their own prescribed clinical and data collection processes, stakeholder preferences and priorities, and staff needs. For example, within the mammography clinics, the screening algorithm had to be embedded in the mammography software due to the clinic flow logistics. However, the mammography software did not interface with the EMR, necessitating a separate, duplicative workflow to ensure appropriate documentation was visible within the EMR. In the GI clinics, while the screening questions and algorithm were embedded within the EMR itself, a streamlined screening tool focusing on CRC only had to be developed due to practitioners’ workflows and needs. The differences between the clinics made data collection, data assimilation, data storage, and data analysis more complex. As a result, we were unable to collect all the desired data points for patients in this program, including extensive demographics data. Standardizing screening tools and methods of data capture and analysis would be essential to ensure more streamlined and efficient processes for future initiatives. Issues such as staffing turnover and competing site-specific priorities often delayed the implementation timeline for the DUAL program.

### Limitations

Given that DUAL data was acquired from multiple clinical sites using different data sources (ie, EMR vs mammography software vs C-SPAN software), we encountered the limitations of each of these platforms, and were unable to capture demographic data for the larger population of the DUAL program. Additionally, since DUAL was housed across different sites that necessitated use of different screening tools, it limited our ability to determine the true denominators, and therefore yields for several of the variables reported. To better contextualize the data, understanding the number of patients across clinics that interfaced with the screening tool and program would be ideal. This number over time between multiple sites over multiple years was a moving target that was unable to be well-defined outside of whom we were able to screen, which does confound interpretation of program reach and uptake. Because we were selecting for patients at increased risk for hereditary cancer prior to offering genetic testing, the overall yield of LS was higher in our population compared to the 1/370 prevalence reported in the general population. Another limitation of this program was the fact that the family history screening questions used were not validated instruments. While the questions were based on validated tools, modifications were made based on stakeholder feedback, and the final questions were not piloted in the target populations. Validated cancer family history screening tools such as PREMM1,2,6 and PREMM5 show sensitivity (72%) and specificity (75%), and also use tailored family history questions to identify individuals with a higher likelihood of LS. However, the trade-off with such tools is that an increase in sensitivity can lead to a drop in specificity.^61^ Given that we used different screening tools across sites due to administrative considerations of the individual clinics, we are unable to truly calculate specificity/sensitivity of this project’s methodology to compare. Lastly, for DUAL, we did not utilize an implementation science framework or other formal construct in the evaluation plan as this was a clinical quality improvement project.

### Future Directions

DUAL is a largely automated and sustainable program, and the population screening measures established with initial grant funding continue today as they were incorporated into clinical data systems (EMR and mammography software). Improved patient access and overall positive outcomes demonstrated the need to the AMC leadership for continued financial support for DUAL personnel. Since the roll-down of the formally funded project, we have continued to monitor downstream outcomes and there are ongoing plans to continue evaluating outcomes through interview of stakeholders, such as clinicians and population in the catchment area through patient advisory councils at the institutions. We continue to analyze barriers identified by the genetics team as well as those identified through query of stakeholders based on our data from this project. Through this evaluation process, we will assess implementation outcomes, such as acceptability of the program as it has historically been conducted and use feedback to fine-tune our operating procedures.

While this population-based cancer family history screening method was initiated in the setting of mammography clinics, GI clinics, and colon cancer screening programs, most aspects of the DUAL program are generalizable and can be replicated in other settings, such as primary care, to reach a broader population. As endorsement for population screening of Tier 1 hereditary cancer predisposition syndromes continues to grow, primary care providers will need to be prepared to provide screening. Implementation of these screening tools will allow general practitioners a simple and efficient way to screen their patients and know when to refer to genetics services. To this end, ongoing conversations with stakeholders about barriers and an upcoming formal needs assessment of stakeholders will further effort towards primary care clinic screening opportunities and capabilities.

Additionally, artificial intelligence platforms are increasingly being deployed to deliver genetic information on a large scale. Tools such as chatbots can increase accessibility to screening for hereditary cancer risk.^62-65^ As noted previously, many individuals, even in underserved or low-income groups, use their phones for medical discussions. As such, an online chatbot-based risk assessment that evaluates for Tier 1 conditions and is easily accessible through a text or email link would be an effective method for identifying high-risk individuals. A web-based chatbot can be easily implemented across populations and clinics without placing a burden on the clinicians themselves or disrupting clinic flows. Furthermore, this process can reduce some of the manual screening and triage navigators perform, making the patient navigation process more efficient than the model used for the DUAL program. While successful implementation of chatbots in cancer screening centers have been reported, the use in underserved populations with possibly lower health literacy needs further study.

## Conclusion

This description of a programmatic experience with implementation of a hereditary cancer screening program in select populations that crosscut different socioeconomic demographics represents an important step in the hereditary cancer population screening discussion. The highlighted success and challenges provide a roadmap for future hereditary cancer populations screening initiatives, and entering into larger population screening arenas is the next frontier to decrease disparities in cancer risk identification and prevention.

## Supplemental Material

Supplemental Material - Implementation of a Population-Based Cancer Family History Screening Program for Lynch Syndrome Click here for additional data file. Supplemental Material for Implementation of a Population-Based Cancer Family History Screening Program for Lynch Syndrome by Sayoni Lahiri, MS, Sara Pirzadeh-Miller, MS, Kelsey Moriarty, MS, and Nisa Kubiliun, MD in Cancer Control

## Appendix

### Abbreviations

AMC: Academic Medical Center
CDC: Centers for Disease Control and Prevention
CGC: Cancer Gene Connect
CGP: Cancer Genetics Program
CH: County Hospital
CPRIT: Cancer Prevention and Research Institute of Texas
CRC: Colorectal cancer
DUAL: Detecting Unaffected Lynch Syndrome
EMR: Electronic medical record
FIT: Fecal immunochemical test
GI: Gastroenterology
HBOC: Hereditary Breast and Ovarian Cancer syndrome
HIPAA: Health Insurance Portability and Accountability Act
LS: Lynch Syndrome
NCCN: National Comprehensive Cancer Network
WHO: World Health Organization
