# Supplementary figures and images for: Implementation of a Population-Based Cancer Family History Screening Program for Lynch Syndrome
Source: Cancer Control. 2023 May 10;30:10732748231175011. doi: 10.1177/10732748231175011 (PMC10185972; doi:10.1177/10732748231175011)

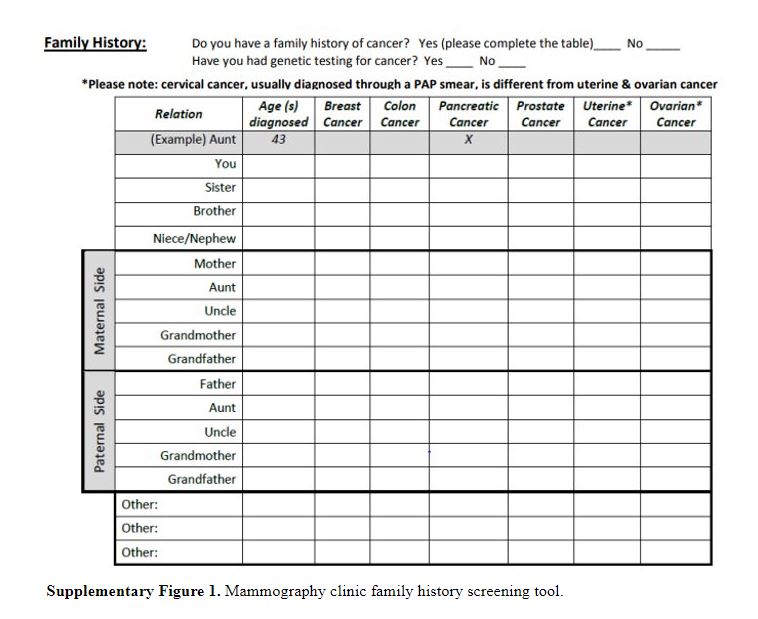

Supplement: Supplemental Material - Implementation of a Population-Based Cancer Family History Screening Program for Lynch Syndrome [file sj-zip-1-ccx-10.1177_10732748231175011.zip › SupFigure1wTitle.JPG]

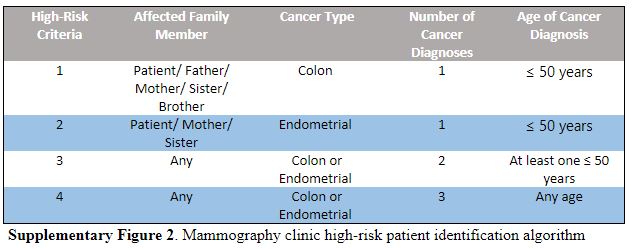

Supplement: Supplemental Material - Implementation of a Population-Based Cancer Family History Screening Program for Lynch Syndrome [file sj-zip-1-ccx-10.1177_10732748231175011.zip › SupFigure2wTitle.JPG]

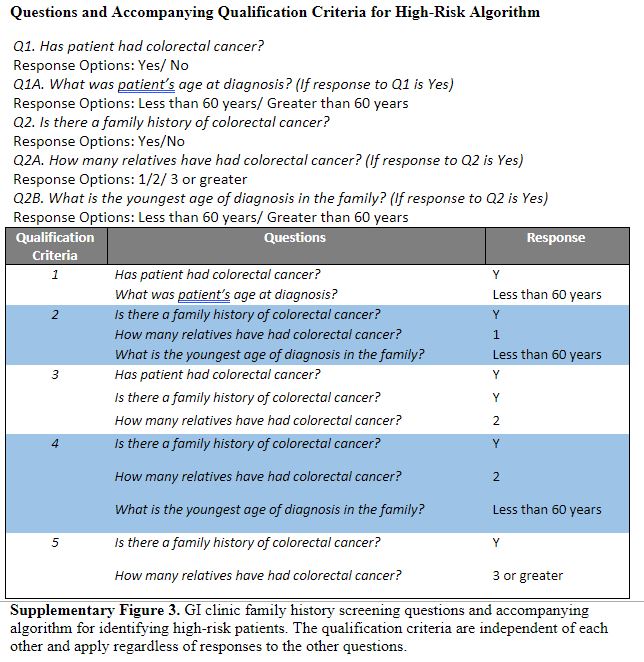

Supplement: Supplemental Material - Implementation of a Population-Based Cancer Family History Screening Program for Lynch Syndrome [file sj-zip-1-ccx-10.1177_10732748231175011.zip › SupFigure3wTitle.JPG]

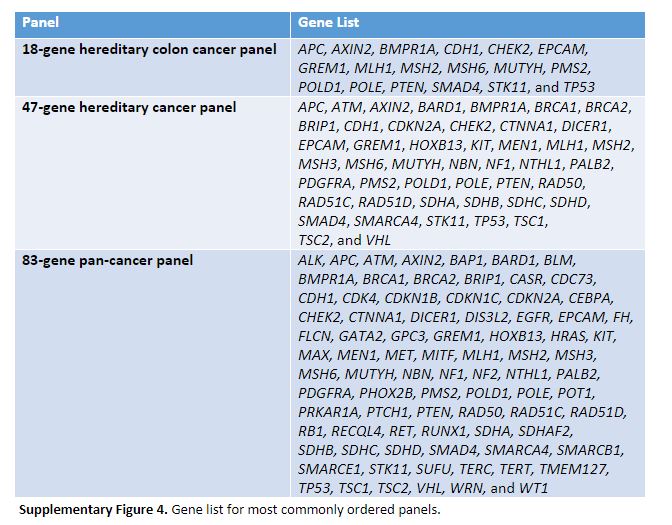

Supplement: Supplemental Material - Implementation of a Population-Based Cancer Family History Screening Program for Lynch Syndrome [file sj-zip-1-ccx-10.1177_10732748231175011.zip › SupFigure4wTitle.JPG]
